# FSH and bone: Comparison between males with central versus primary hypogonadism

**DOI:** 10.3389/fendo.2022.939897

**Published:** 2022-08-05

**Authors:** Luca Giovanelli, Richard Quinton, Biagio Cangiano, Stefano Colombo, Luca Persani, Marco Bonomi, Iacopo Chiodini

**Affiliations:** ^1^ Department of Medical Biotechnology and Translational Medicine, University of Milan, Milan, Italy; ^2^ Department of Endocrine and Metabolic Medicine, IRCCS Istituto Auxologico Italiano, Milan, Italy; ^3^ Department of Endocrinology, Diabetes & Metabolism, Newcastle-upon-Tyne Hospitals, Newcastle upon Tyne, United Kingdom; ^4^ Translational & Clinical Research Institute, University of Newcastle-upon-Tyne, Newcastle upon Tyne, United Kingdom

**Keywords:** follicle-stimulating hormone, male hypogonadism, hypergonadotropic, hypogonadotropic, bone metabolism, male osteoporosis

## Abstract

**Objective:**

Experimental studies proposed a direct effect of follicle-stimulating hormone (FSH) on the skeletal metabolism, but results of human studies mainly conducted in females are controversial. The present study aims to investigate the possible role of FSH excess in male bone health, by comparing for the first time primary and central hypogonadism.

**Design and Methods:**

119 men were enrolled in this cross-sectional observational study at the time of the first diagnosis of hypogonadism. All participants had spontaneous pubertal development. Regarding patients with hypergonadotropic hypogonadism (Hyper-H), Klinefelter syndrome (KS) patients were distinguished from the other forms (non-KS-Hyper-H) based on the onset of FSH elevation. Bone mineral density (BMD) at both lumbar spine (LS) and femoral neck (FN), as well as the prevalence of morphometric vertebral fractures (VFx), were assessed.

**Results:**

Across the whole cohort, higher LS and FN BMD were associated with older age at diagnosis and higher body mass index (BMI), respectively. After adjusting for potential confounders (age at diagnosis, BMI, smoking habits, degree of hypogonadism defined by calculated free testosterone, and 25OH vitamin D levels), non-KS-Hyper-H patients showed significantly lower LS BMD and tended to show lower FN BMD values, as compared to those with hypogonadotropic hypogonadism (Hypo-H). In KS men, LS BMD was significantly lower than in those with non-KS-Hyper-H. No significant differences in the prevalence of VFx were found between the groups.

**Conclusions:**

These findings suggest a potential negative effect of FSH excess on the male bone mass, especially at spine. The duration of high FSH levels may also contribute to these findings.

## Introduction

Follicle-stimulating hormone (FSH) is a heterodimeric glycoprotein secreted by the anterior pituitary gland. It consists of a hormone-specific β subunit non-covalently associated with the α subunit that is also part of other glycoprotein hormones (1). FSH activity is mediated *via* FSH receptor (FSHR), which belongs to the large superfamily of 7-transmembrane-spanning (7TM) G protein-coupled receptors (GPCR) (2). The binding of FSH to its receptor results in the activation of adenylyl cyclase and, thus, in the initiation of the cAMP signalling cascade (3). Gonads are the main target tissue of FSH, which in females is responsible for follicular development and oestrogen production and, in males, regulates Sertoli cells proliferation and function and promotes spermatogenesis (4).

Over the last few decades, FSH has been also shown to stimulate bone resorption (5–9), by binding to its specific receptor on osteoclasts. In fact, a polyclonal antibody generated against FSHβ proved to be able to block osteoclastogenesis *in vitro* and, when injected into ovariectomized mice, to attenuate bone loss (10, 11). Further, FSH may negatively regulate osteoblast differentiation from mesenchymal stem cells.

In keeping with these findings, several observational studies involving large cohorts of perimenopausal and postmenopausal women consistently reported an association between raised FSH values and increased bone turnover as well as reduced bone mineral density (BMD) (12–14). A potential role of polymorphisms in the FSH/FSHR system has been hypothesized in this setting (15). Notably, in women of reproductive age, hypergonadotropic amenorrhea was found to be associated with greater bone loss than hypothalamic amenorrhea, irrespective of serum oestradiol concentrations (16). FSH levels also appear to be a good negative predictor of BMD in patients on sex hormone replacement therapy (HRT) (17). By contrast, two interventional studies failed to detect any causal relationship between FSH and bone metabolism (17–19).

With regards to males, the existing literature is sparse and conflicting. On the one hand, according to previous studies involving osteoporotic men (20), patients with Klinefelter syndrome (KS) (3, 21), and those on androgen deprivation therapy for prostate cancer (22), FSH appears to have a detrimental effect on the male bone. On the other hand, a recent longitudinal study found no evidence for an association between baseline FSH levels and changes in bone mass (23). Moreover, bone health was not found to be impaired in a cohort of men with infertility, high FSH but normal testosterone (T) levels (24, 25). Finally, no significant change was detected in bone turnover markers in men after suppressing FSH levels (26).

However, all these studies present some limitations, particularly relating to selection and/or confounding bias, as well as short duration and lack of baseline assessment. Therefore, on balance, the putative role of FSH as a direct modulator of skeletal physiology remains matter of debate. Intriguingly, men with primary versus central hypogonadism, both presenting with low T but with differing FSH values, might represent a novel attractive study model in this context.

The present study aims to investigate the possible association of FSH excess with male osteoporosis, by assessing BMD and prevalence of fragility fractures in men with primary hypogonadism (high FSH levels) compared to those with central hypogonadism (low/normal FSH levels).

## Materials and Methods

One hundred and nineteen men, consecutively referred to the Hypogonadism Clinic both of Istituto Auxologico Italiano (IAI, Milan) and Newcastle upon Tyne Hospitals (NHS Foundation Trust) for clinical suspicion - later confirmed - of hypogonadism, were enrolled in this cross-sectional observational study. Diagnosis of hypogonadism was based on the finding of consistently low T levels (total T < 8 nmol/L or < 12 nmol/L in the presence of calculated free T < 225 pmol/L).

**Figure 1:**
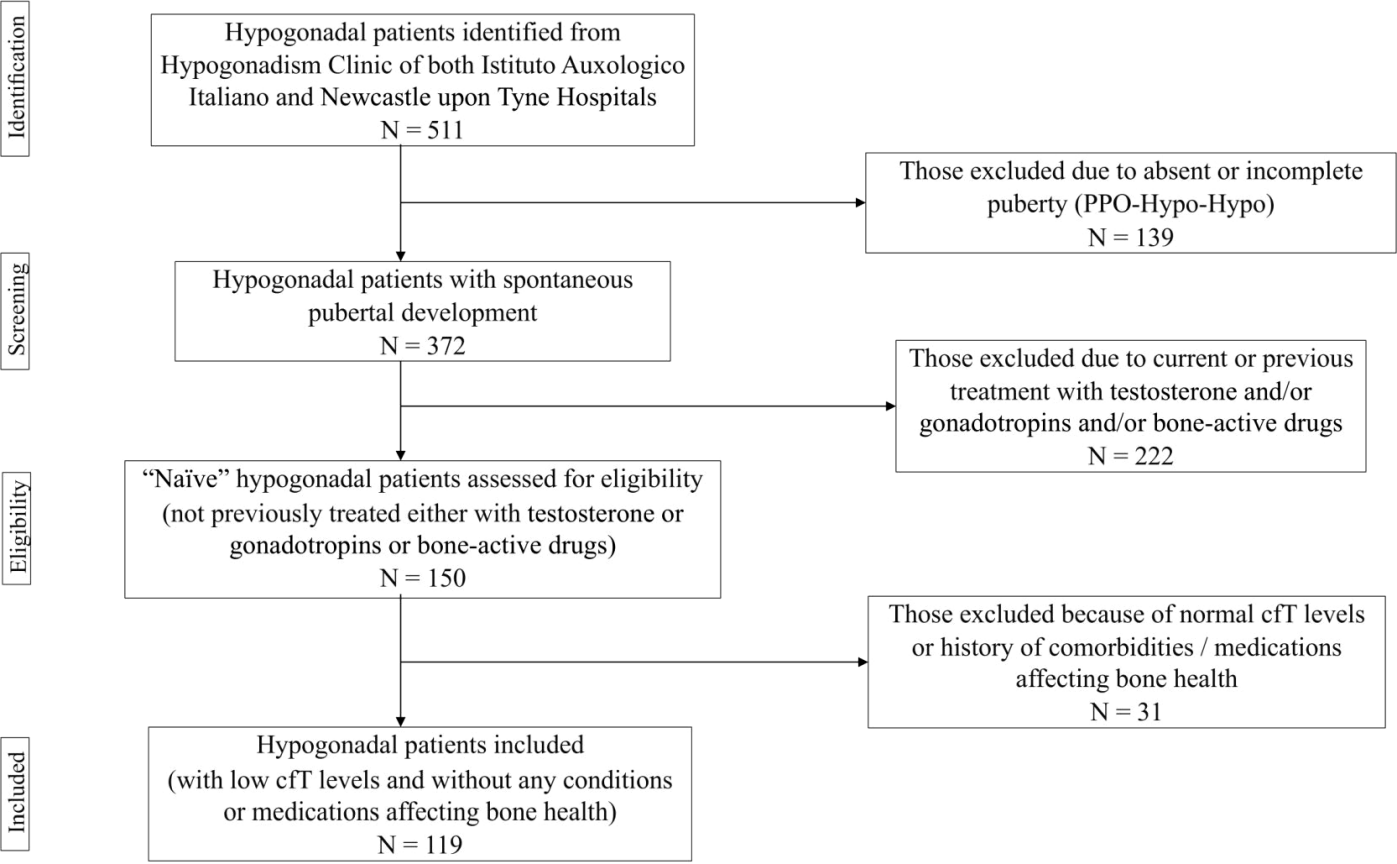
Flow chart used for selecting participants. PPO-Hypo-Hypo, pre-pubertal onset hypogonadotropic hypogonadism; cfT, calculated free testosterone.

The project was approved by the Institutional Review Board of IAI (05C622_2016). Recruitment lasted 18 months. Subjects fulfilling one or more of the following criteria were excluded: I) absent puberty; II) current or previous treatment with testosterone and/or gonadotropins, antiresorptive and/or anabolic agents, or any other medication impacting on bone metabolism (e.g., glucocorticoids, anticonvulsants, thiazolidinediones); III) comorbidities that may affect skeletal health, such as hyperthyroidism, hyperparathyroidism, rheumatoid arthritis, malabsorption, alcohol abuse, chronic kidney or liver disease. The flow-chart used to select participants is depicted in **Figure 1**. Basically, we decided to include only naïve patients with spontaneous pubertal development. In fact, both pubertal delay and previous testosterone replacement therapy (TRT) could be relevant confounders due to their respectively detrimental and beneficial effects on skeleton (27–29).

Causes of hypogonadism and clinical characteristics of the whole cohort are summarized in **Table 1** and **Table 2**, respectively. Patients were classified as affected either with hypogonadotropic hypogonadism (Hypo-H) or hypergonadotropic hypogonadism (Hyper-H). As far as the latter is concerned, Klinefelter syndrome (KS) patients were analysed separately from the other forms of Hyper-H (non-KS-Hyper-H) according to the onset of FSH elevation, which occurred at the time of pubertal development (several years prior to T deficiency onset) and later in adult life (coinciding with T deficiency onset), respectively. In KS patients, the age at diagnosis refers to the first finding of low T levels.

**Table 1:** Etiology of hypogonadism.

Hypergonadotropic hypogonadism (n=72)		Hypogonadotropic hypogonadism (n=47)
Klinefelter syndrome (n=46)	Testicular trauma/torsion (n=3)	AO-nCHH (n=40)
	Orchitis/Epididymitis (n=2)	LOH (metabolic syndrome-related) (n=5)
	Cryptorchidism (n=2)	Pituitary macroadenoma (n=2)
	Testicular cancer treated with unilateral orchiectomy (n=2)	
	Autoimmune (APS) (n=1)	
	LOH (age-related)/Idiopathic (n=16)	

AO-nCHH, adult onset-normosmic congenital hypogonadotropic hypogonadism; LOH, late onset hypogonadism; APS, autoimmune polyglandular syndrome.

**Table 2:** Clinical characteristics of the whole cohort and comparison between patients with hypergonadotropic hypogonadism (Hyper-H) and those with hypogonadotropic hypogonadism (Hypo-H).

	All subjects(n=119)	Hyper-H(n=72)	Hypo-H(n=47)	p
**Age at diagnosis (years)**	45.5 ± 14.6 (17-80)	44.9 ± 15.9 (18-80)	46.3 ± 12.5 (17-68)	0.6
**BMI (kg/m^2^)**	26.9 ± 5.2 (19.1-44.4)	26.1 ± 4.5 (19.1-37.4)	28.1 ± 6.0 (19.3-44.4)	**0.04**
**Overweight/obesity**	66 (55.5)	34 (47.2)	32 (68.1)	**0.02**
**LS Z-score (SD)**	-0.9 ± 1.3 (-3.4 – 3.2)	-1.0 ± 1.4 (-3.2 – 3.2)	-0.7 ± 1.1 (-3.4 – 1.8)	**0.01^§^**
**FN Z-score (SD)**	-0.7 ± 1.0 (-2.8 – 1.8)	-0.7 ± 1.1 (-2.7 – 1.6)	-0.6 ± 1.0 (-2.8 – 1.8)	0.07^§^
**VFx**	22 (18.5)	14 (19.4)	8 (17.0)	0.6
**Other fragility Fx**	4 (3.4)	2 (2.8)	2 (4.3)	0.5
**aCa (mg/dl)**	9.5 ± 0.4 (8.2-10.4)	9.5 ± 0.4 (8.4-10.4)	9.4 ± 0.4 (8.2-10.1)	0.06
**P (mg/dl)**	3.3 ± 0.6 (2.5-4.7)	3.3 ± 0.6 (2.5-4.7)	3.3 ± 0.6 (2.5-4.4)	0.9
**ALP (IU/L)**	78.3 ± 28.7 (42-199)	78.0 ± 28.5 (42-199)	78.9 ± 29.3 (43-182)	0.9
**25OHD (ng/ml)**	25.5 ± 12.6 (3.0-71.5)	27.8 ± 12.2 (8.0-71.5)	22.4 ± 12.7 (3.0-53.1)	**0.04**
**FSH (IU/L)**	26.0 ± 23.0 (0.3-96.5)	40.5 ± 18.2 (10.3-96.5)	3.6 ± 2.1 (0.3-7.6)	**<0.005**
**LH (IU/L)**	15.3 ± 13.3 (0.3-54)	23.6 ± 10.7 (11.6-54.0)	2.5 ± 1.5 (0.3-6.5)	**<0.005**
**cfT (pmol/L)**	113.2 ± 51.3 (5-224)	119.0 ± 53.8 (16-224)	104.0 ± 46.2 (5-193)	0.1
**T2D/IFG**	23 (19.3)	12 (16.7)	11 (23.4)	0.4
**AH**	24 (20.2)	12 (16.7)	12 (25.5)	0.2
**Dyslipidaemia**	22 (18.5)	11 (15.3)	11 (23.4)	0.3
**Smoke (>5 cig/d)**	16 (13.4)	9 (12.5)	7 (14.9)	0.7

Data are expressed as mean value ± SD (range) or absolute number (percentage). **^§^** after adjusting for age at diagnosis, BMI, cfT and 25OHD levels by means of General Linear Model analysis. BMI, body mass index; T2D, type 2 diabetes; IFG, impaired fasting glucose; AH, arterial hypertension; cfT, calculated free testosterone; aCa, albumin-adjusted calcium; P, phosphorus; ALP, alkaline phosphatase; 25OHD, 25OH vitamin D; LS, lumbar spine; FN, femoral neck; VFx, vertebral fractures.

Bold values: statistically significant (p<0.05).

All the participants were studied at the time of the first diagnosis of hypogonadism. Relevant patient data were prospectively assembled as part of routine clinical practice based on the delivery of good clinical care.

Personal and family history was investigated, with focus on previous fragility fractures as well as any conditions or drugs that may affect bone health. Regarding smoking habits, a daily consumption of more than 5 cigarettes was considered as a risk threshold (30).

Weight and height were measured, and body mass index (BMI) was calculated. Waist circumference (WC), arm span (AS), Tanner stage, testicular volume, blood pressure (BP) and heart rate (HR) were assessed, too.

Bone mineral density at both lumbar spine (LS, L1-L4) and femoral neck (FN) was measured using dual-energy X-ray absorptiometry (DEXA, Hologic or Lunar), and expressed as standard deviation (SD) units in relation to the young (*t*-score) and age-matched (*z*-score) reference healthy population.

The prevalence of vertebral fractures (VFx) was evaluated by performing vertebral morphometry on conventional thoraco-lumbar-sacral spine radiographs (T5-L4, in lateral and anteroposterior projections). The semiquantitative visual assessment previously described by Genant et al. (31) was employed.

Besides, serum calcium (Ca), albumin, phosphorus (P), alkaline phosphatase (ALP), glucose and lipid profile were determined using standard techniques. 25-hydroxy-vitamin D levels (25OHD) levels were measured by means of Elecsys Roche assay. Albumin-adjusted calcium (aCa) levels were obtained. Free testosterone (cfT) levels also were calculated from total testosterone (tT), albumin and sex hormone-binding globulin (SHBG) using the Vermeulen equation (32).

As per the *BonesGone project*, these data were extracted from case records and collated in fully anonymised format prior to statistical analysis. Written consent was obtained from each patient or subject after full explanation of the purpose and nature of all procedures used, unless these formed part of everyday clinical care.

### Statistical Analysis

Statistical analysis was performed using SPSS version 27.0 statistical package (SPSS Inc, Chicago, IL). The normality of distribution for continuous variables was tested using Kolmogorov–Smirnov test. Data were expressed as median (range) for non-normally distributed continuous variables or as mean ± SD for normally distributed variables, whereas as absolute and relative frequencies for categorical variables.

Continuous variables were compared between Hyper-H and Hypo-H (**Table 2**) using one-way Student t test or Mann–Whitney U test, as appropriate. One way analysis of variance with Bonferroni *post hoc* analysis was performed to compare the continuous variables among KS, non-KS-Hyper-H and Hypo-H (**Table 3**). Categorical variables were compared using X^2^ or Fisher’s Exact test, as appropriate. General Linear Model (GLM) was employed to assess the independent associations between BMD and the variables that were found to be different among groups and those known to be relevant for bone health.

**Table 3:** Comparison among patients with Klinefelter syndrome (KS), with other forms of hypergonadotropic hypogonadism (non-KS-Hyper-H) and with hypogonadotropic hypogonadism (Hypo-H).

	KS(n=46)	non-KS-Hyper-H(n=26)	Hypo-H(n=47)
**Age at diagnosis (years)**	39.2 ± 13.2 (18-67)	55.2 ± 15.2 * (26-80)	46.3 ± 12.5 * ˜ (17-68)
**BMI (kg/m^2^)**	26.3 ± 4.9 (19.1-37.4)	25.6 ± 3.8 (19.4-35.0)	28.1 ± 6.0 **˜** (19.3-44.4)
**Overweight/obesity**	24 (52.2)	10 (38.5)	32 (68.1) **˜**
**LS Z-score (SD)**	-1.3 ± 1.1 (-3.2 – 1.5)	-0.8 ± 1.7 * (-3.1 – 3.2)	-0.7 ± 1.1 **^§^** (-3.4 – 1.8)
**FN Z-score (SD)**	-0.7 ± 1.1 (-2.7 – 1.6)	-0.7 ± 1.0 (-2.1 – 1.6)	-0.6 ± 1.0 (-2.8 – 1.8)
**VFx**	9 (19.6)	5 (19.2)	8 (17.0)
**Other fragility Fx**	2 (4.3)	0 (0)	2 (4.3)
**aCa (mg/dl)**	9.5 ± 0.4 (8.4-10.3)	9.5 ± 0.5 (8.7-10.4)	9.4 ± 0.4 (8.2-10.1)
**P (mg/dl)**	3.3 ± 0.6 (2.5-4.7)	3.4 ± 0.5 (2.6-4.2)	3.3 ± 0.6 (2.5-4.4)
**ALP (IU/L)**	80.9 ± 31.5 (42-199)	73.4 ± 22.9 (43-135)	78.9 ± 29.3 (43-182)
**25OHD (ng/ml)**	25.8 ± 13.4 (9.0-71.5)	30.5 ± 9.9 (8.0-47.2)	22.4 ± 12.7 **˜** (3.0-53.1)
**FSH (IU/L)**	40.4 ± 17.4 (16.6-96.5)	40.7 ± 20.0 (10.3-85.0)	3.6 ± 2.1 * ˜ (0.3-7.6)
**LH (IU/L)**	24.0 ± 10.0 (12.1-54)	23.0 ± 12.1 (11.6-51.0)	2.5 ± 1.5 * ˜ (0.3-6.5)
**cfT (pmol/L)**	112.7 ± 57.3 (16-224)	130.3 ± 45.9 (55-210)	104.0 ± 46.2 **˜** (5-193)
**T2D/IFG**	6 (13.0)	6 (23.1)	11 (23.4)
**AH**	6 (13.0)	6 (23.1)	12 (25.5)
**Dyslipidaemia**	5 (10.9)	6 (23.1)	11 (23.4)
**Smoke (>5 cig/d)**	8 (17.4)	1 (3.8)	7 (14.9)

Data are expressed as mean value ± SD (range) or absolute number (percentage). *p<0.05 vs KS; **˜**p<0.05 vs non-KS-Hyper-H; **^§^** p<0.05 vs non-KS-Hyper-H after adjusting for age at diagnosis, BMI, cfT, 25OHD levels and smoking habits by means of General Linear Model analysis. BMI, body mass index; T2D, type 2 diabetes; IFG, impaired fasting glucose; AH, arterial hypertension; cfT, calculated free testosterone; aCa, albumin-adjusted calcium; P, phosphorus; ALP, alkaline phosphatase; 25OHD, 25OH vitamin D; LS, lumbar spine; FN, femoral neck; VFx, vertebral fractures.

P values <0.05 were considered statistically significant.

## Results

### Comparison between patients with hypergonadotropic hypogonadism (Hyper-H) and those with hypogonadotropic hypogonadism (Hypo-H)

As compared with Hypo-H patients, those with Hyper-H had lower BMI (26.1 ± 4.5 vs 28.1 ± 6.0 kg/m^2^; p=0.04) and overweight/obesity prevalence (47.2% vs 68.1%; p=0.02) with no significant differences in age, prevalence of type 2 diabetes (T2D) and smoking habits (**Table 2**).

Notably, in the Hyper-H group, 25OHD concentrations were higher (27.8 ± 12.2 vs 22.4 ± 12.7 ng/ml; p=0.04), and cfT (119.0 ± 53.8 vs 104.0 ± 46.2 pmol/L; p=0.1) as well as aCa levels (9.5 ± 0.4 vs 9.4 ± 0.4 mg/dl; p=0.06) tended to be higher as compared with Hypo-H group, while the BMD values and the prevalence of fragility fractures were comparable between the groups.

However, after adjusting for age at diagnosis, BMI, cfT and 25OHD levels by means of GLM analysis, LS BMD was significantly lower (-1.0 ± 1.4 vs -0.7 ± 1.1 SD; p=0.01), and FN BMD tended to be lower (-0.7 ± 1.1 vs -0.6 ± 1.0 SD; p=0.07) in Hyper-H patients than in Hypo-H ones.

**Figure 2:**
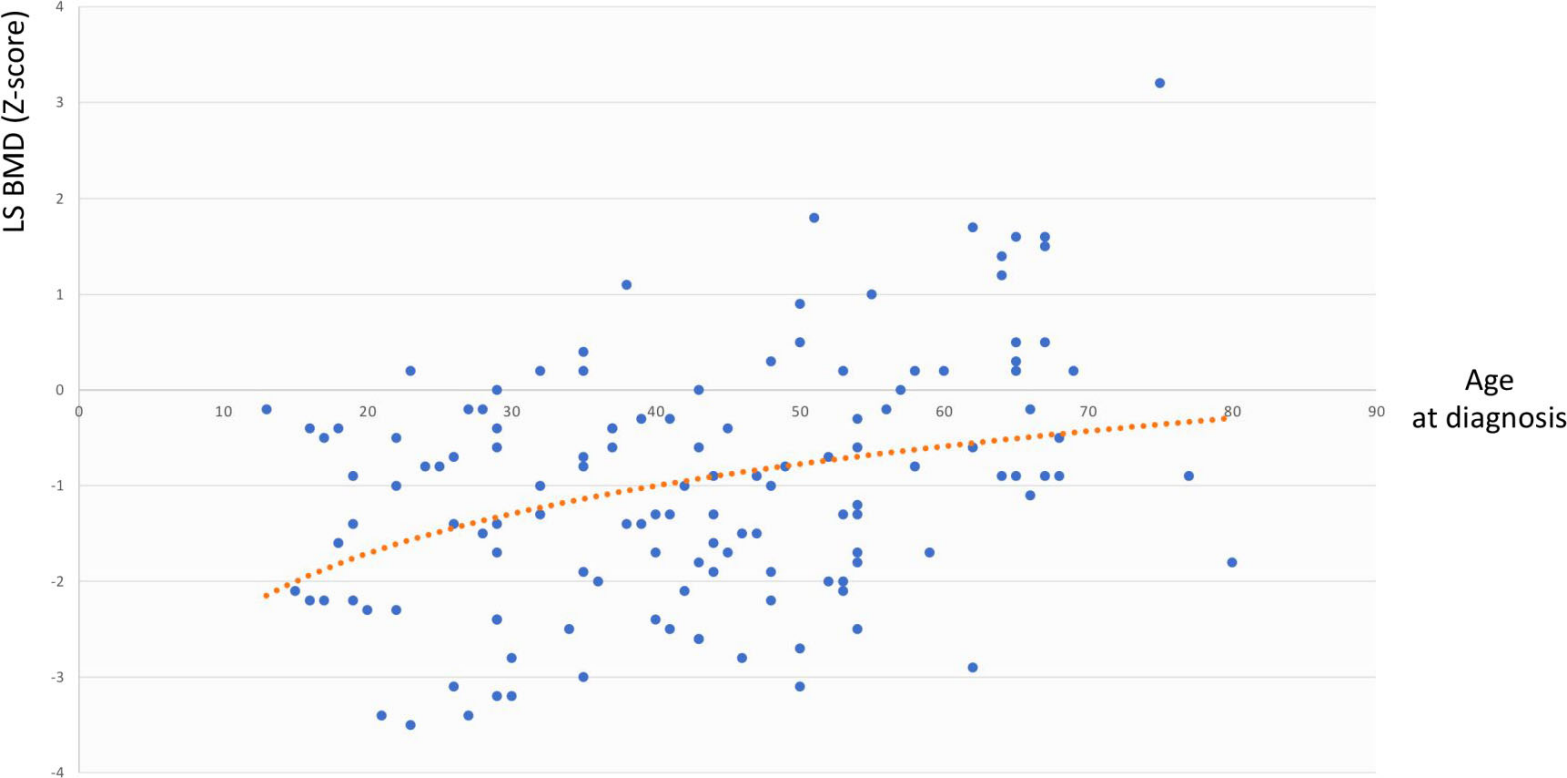
Association between LS BMD and age at diagnosis. LS BMD, lumbar spine bone mineral density.

**Figure 3:**
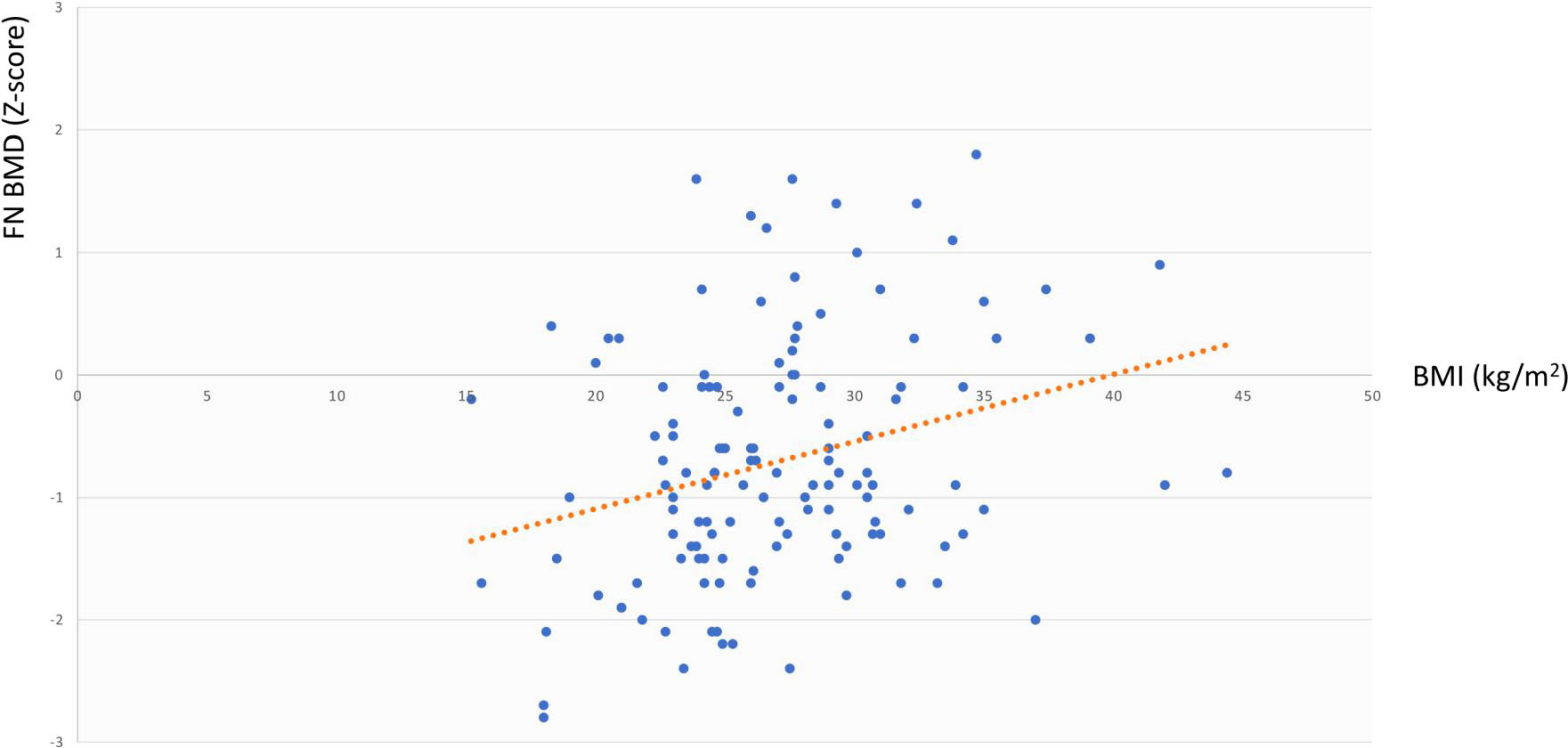
Association between FN BMD and BMI. FN BMD, femoral neck bone mineral density; BMI, body mass index.

The same analysis showed that in the whole cohort LS BMD was directly associated with age at diagnosis (r=0.344, p=0.003) but not with BMI, cfT and 25OHD levels, whereas FN BMD was associated with BMI (r=0.291, p=0.02) but not with age at diagnosis, cfT and 25OHD levels. The bivariate associations between LS BMD and age at diagnosis, and between FN BMD and BMI are depicted in **Figure 2** and **Figure 3**, respectively.

### Comparisons among patients with Klinefelter syndrome (KS), with non-KS-hypergonadotropic hypogonadism (non-KS-Hyper-H) and with hypogonadotropic hypogonadism (Hypo-H)

In order to evaluate the possible influence of the duration of FSH excess, we compared KS patients with non-KS-Hyper-H patients (**Table 3**). The former were diagnosed at a younger age (39.2 ± 13.2 vs 55.2 ± 15.2 years; p<0.001) and displayed significantly lower BMD values at LS (-1.3 ± 1.1 vs -0.8 ± 1.7 SD; p=0.01) as compared with the latter ones, while no differences were found between the two groups in terms of smoking habits, cfT and 25OHD levels, as well as FN BMD and presence of fragility fractures.

We also compared the clinical characteristics between non-KS-Hyper-H and Hypo-H ones (**Table 3**). This analysis did not reveal any statistically significant difference as far as the prevalence of fragility fractures was concerned, while non-KS-Hyper-H patients had higher cfT (130.3 ± 45.9 vs 104.0 ± 46.2 pmol/L; p=0.03) and 25OHD levels (30.5 ± 9.9 vs 22.4 ± 12.7 ng/ml; p=0.02), as well as lower BMI (25.6 ± 3.8 vs 28.1 ± 6.0 kg/m^2^; p=0.04) and prevalence of overweight/obesity (38.5% vs 68.1%; p=0.04) than Hypo-H patients. After adjusting for age at diagnosis, BMI, cfT, 25OHD levels and smoking habits, LS BMD was significantly lower (-0.8 ± 1.7 vs -0.7 ± 1.1 SD; p=0.01), and FN BMD tended to be lower (-0.7 ± 1.0 vs -0.6 ± 1.0; p=0.07) in non-KS-Hyper-H than in Hypo-H men.

Besides, in the whole sample of hypogonadal men with adult-onset FSH elevation, LS BMD was directly associated with both age at diagnosis (r=0.360, p=0.002) and overweight/obesity prevalence (r=0.338, p=0.003), but not with smoking habits, cfT and 25OHD levels, whilst FN BMD tended to be associated with age at diagnosis (r=0.320, p=0.06) but not with overweight/obesity prevalence, smoking habits, cfT and 25OHD levels.

## Discussion

This is the first study comparing men with primary and central hypogonadism to better delineate the role of FSH in the male bone health. Notably, such findings indicate a potential negative effect of FSH excess on the male bone mass, especially at spine, either in the whole cohort or within the subset of hypogonadal patients with adult-onset FSH elevation. The duration of high FSH levels may also play a part in this setting, as revealed by the comparison between KS and non-KS-Hyper-H men.

Indeed, several findings support the FSH fostering role on bone loss in males. For instance, in KS patients neither T levels nor polymorphisms in the androgen receptor (AR) gene - consisting of a variable number of CAG repeats which modulate its sensitivity - are associated with osteoporosis (21), thus pointing to a potential impact of high FSH levels. With regards to AR gene polymorphisms, TRT has been seen to induce greater BMD improvement in men with post-surgical hypogonadotropic hypogonadism harbouring shorter CAG tract (33). Importantly, elevated FSH levels could compound the association between increased bone resorption and metastatic bone invasion observed in patients on androgen deprivation therapy for prostate cancer (22). Moreover, a negative correlation between FSH concentrations and lumbar/femoral BMD was found in 156 men from a case-control study (20). In contrast, there was no evidence for an association between baseline FSH levels and changes in bone mass in a recent longitudinal observational study of Icelandic older adults (23). Likewise, in a group of 137 middle aged men with idiopathic spermatogenic failure, who are, hence, exposed to high FSH in the presence of normal T levels, bone health was not found to be impaired (24). However, this latter study presents the following limitations: a) exclusion of men with explained infertility; b) potential non-response bias and lack of baseline BMD measurements (24). Similar data were observed in another cohort of men with idiopathic infertility or KS (25). Lastly, in a sub-analysis of the Hypogonadism in Men study, Uihlein and colleagues detected no significant change in bone turnover markers after suppressing FSH by means of a gonadotropin-releasing hormone (GnRH) analog for 16 weeks, in comparison with controls receiving placebo (26). Nevertheless, only eugonadal subjects were enrolled, and duration and/or magnitude of FSH reduction may have been insufficient (26).

In our project, after adjusting for potential confounders, Hyper-H patients showed lower LS BMD, and tended to show lower FN BMD levels, as compared to Hypo-H patients (either in the whole cohort or within the subset of hypogonadal patients with adult-onset FSH elevation). Since we enrolled only patients naïve of any treatment or condition possibly interfering with BMD, these findings give a strong support to the negative effect of FSH excess on the male bone mass. Trabecular bone seems to be affected to a greater extent, in keeping with what is observed in perimenopausal women and, more generally, in high-turnover osteoporosis. In fact, FSH elevation may partly explain the decline in bone density that occurs during the early menopausal transition when oestrogen levels are still well-preserved (34, 35).

Furthermore, in KS patients LS BMD values were lower than in the other Hyper-H ones. Notably, this may reflect the effect of long-lasting FSH excess (starting from puberty) on trabecular bone. Indeed, it is worth noting that hypogonadism can just partly justify bone fragility in KS (36, 37), and additional factors should be taken into account, such as decreased vitamin D and insulin-like factor 3 (INSL3) concentrations (37, 38), sarcopenia (36) and genetic factors (21, 39). Long-lasting elevated FSH - not fully normalised by TRT - might hence represent a further contributor to osteoporosis in this condition.

By carrying out the GLM analysis in the whole cohort, we found LS BMD and FN BMD to be independently and directly associated with age at diagnosis and BMI, respectively (**Figures 2**, **3**). The same analysis, when restricted to patients with adult-onset FSH elevation, confirmed only partially such results, probably because of the smaller sample size. Overall, the relationship between BMD and age at diagnosis could be simply related to the progressively longer timeframe available for bone accrual before the occurrence of hypogonadism. On the other side, the mechanisms that may account for the direct correlation between BMD and BMI, include increased loading (especially at hip) along with raised levels of leptin and oestrogen due to increased expression of aromatase in the adipose tissue of obese subjects (40). Under some circumstances, obesity (especially when accompanied by T2D and hypovitaminosis D) can impair bone quality, even though bone density may be normal or even higher than normal (41, 42).

As expected (28), Hypo-H patients showed higher BMI and overweight/obesity prevalence compared with Hyper-H ones. Although just few participants were diagnosed with metabolic syndrome-related late-onset hypogonadism (LOH), overweight/obesity might indeed play a role also among those labelled as adult-onset-congenital hypogonadotropic hypogonadism (**Table 1**). It is in fact challenging to distinguish between these two forms of central hypogonadism (43). In this regard, recent data have suggested a genetic predisposition underlying LOH as well (44).

Our project also presents some limitations. Firstly, the final sample size of patients was limited but balanced by the rigorous design of the study that excluded many hypogonadal patients (**Figure 1**). Although the average duration of disease was not available, we did not discern any remarkable differences between groups. Moreover, some patients have been probably commenced on vitamin D supplements some time before being enrolled, which may have influenced 25OHD circulating levels but not BMD. Lastly, the cross-sectional design allowed to find associations but not to demonstrate a link of causality. Further longitudinal studies, involving patients on TRT, are required.

Crucially, the worldwide incidence of fragility fractures has been exponentially growing, resulting in significantly impaired quality of life and increased mortality and social burden. Although up to 30-40% of all fragility fractures affect men (45), male osteoporosis remains to date underdiagnosed and undertreated. Therefore, finding novel risk factors and mediators for male bone fragility could guide earlier identification of high-risk subjects, with a view to ultimately reducing the huge public health burden related to fractures. Further, these data prompt the future deployment of FSH-blocking agents in order to uncouple the two phases of bone turnover and thus achieve an anabolic net effect (46, 47). Another promising strategy might be FSHβ vaccine (48). The data also support early intervention with testosterone treatment in males with compensated hypogonadism (raised LH & FSH with normal T level), including KS patients, rather than waiting for serum T levels to decline below a given threshold.

## Conclusions

The present study suggests a negative effect of FSH excess on bone mass, especially at spine, in men with hypergonadotropic hypogonadism. The duration of high FSH levels may also have a role in this context. Overall, in hypogonadal males, trabecular bone seems to be associated with age, whereas BMI may influence to a greater extent cortical bone.

## Data availability statement

The datasets presented in this study can be found in online repositories. The names of the repository/repositories and accession number(s) can be found below: 10.5281/zenodo.6472089.

## Ethics statement

The studies involving human participants were reviewed and approved by the Ethics Committee of Istituto Auxologico Italiano. Written informed consent to participate in this study was provided by the participants’ legal guardian/next of kin.

## Author contributions

IC, MB, and LG contributed to conception and design of the study. LG organized the database. LG, MB, RQ, LP, and BC contributed to recruit patients. SC helped to collect data. IC and LG performed the statistical analysis. LG wrote the first draft and sections of the manuscript. IC, MB, RQ, and LP contributed to manuscript revision. All authors read and approved the submitted version.

## Funding

This study was partially supported by Ricerca Corrente funds from IRCCS Istituto Auxologico Italiano: (grant numbers: O5C202_2012 and 05C622_2016).

## Conflict of interest

The authors declare that the research was conducted in the absence of any commercial or financial relationships that could be construed as a potential conflict of interest.

## Publisher’s note

All claims expressed in this article are solely those of the authors and do not necessarily represent those of their affiliated organizations, or those of the publisher, the editors and the reviewers. Any product that may be evaluated in this article, or claim that may be made by its manufacturer, is not guaranteed or endorsed by the publisher.

## References

[1] Das N Kumar TR. Molecular regulation of follicle-stimulating hormone synthesis, secretion and action. J Mol Endocrinol (2018) 60:R131–55. doi: 10.1530/JME-17-0308 PMC5851872 29437880

[2] Bonomi M Persani L. Modern methods to investigate the oligomerization of glycoprotein hormone receptors (TSHR, LHR, FSHR). Methods Enzymol (2013) 521:367–83. doi: 10.1016/B978-0-12-391862-8.00020-X 23351750

[3] Bonomi M Busnelli M Persani L Vassart G Costagliola S. Structural differences in the hinge region of the glycoprotein hormone receptors: Evidence from the sulfated tyrosine residues. Mol Endocrinol (2006) 20:3351–63. doi: 10.1210/me.2005-0521 16901970

[4] Mills EG Yang L Nielsen MF Kassem M Dhillo WS Comninos AN. The relationship between bone and reproductive hormones beyond estrogens and androgens. Endocr Rev (2021) 42:691–719. doi: 10.1210/endrev/bnab015 33901271 PMC8599211

[5] Sun L Peng Y Sharrow AC Iqbal J Zhang Z Papachristou DJ. FSH directly regulates bone mass. Cell (2006) 125:247–60. doi: 10.1016/j.cell.2006.01.051 16630814

[6] Iqbal J Sun L Kumar TR Blair HC Zaidi M. Follicle-stimulating hormone stimulates TNF production from immune cells to enhance osteoblast and osteoclast formation. Proc Natl Acad Sci USA (2006) 103:14925–30. doi: 10.1073/pnas.0606805103 PMC1595452 17003115

[7] Robinson LJ Tourkova I Wang Y Sharrow AC Landau MS Yaroslavskiy BB. FSH-receptor isoforms and FSH-dependent gene transcription in human monocytes and osteoclasts. Biochem Biophys Res Commun (2010) 394:12–7. doi: 10.1016/j.bbrc.2010.02.112 PMC2856932 20171950

[8] Sun L Zhang Z Zhu LL Peng Y Liu X Li J. Further evidence for direct pro-resorptive actions of FSH. Biochem Biophys Res Commun (2010) 394:6–11. doi: 10.1016/j.bbrc.2010.02.113 20171951 PMC3144627

[9] Wu Y Torchia J Yao W Lane NE Lanier LL Nakamura MC. Bone microenvironment specific roles of ITAM adapter signaling during bone remodeling induced by acute estrogen-deficiency. PloS One (2007) 2(7):e586. doi: 10.1371/journal.pone.0000586 17611621 PMC1895921

[10] Zhu LL Blair H Cao J Yuen T Latif R Guo L. Blocking antibody to the β-subunit of FSH prevents bone loss by inhibiting bone resorption and stimulating bone synthesis. Proc Natl Acad Sci USA (2012) 109:14574–9. doi: 10.1073/pnas.1212806109 PMC3437842 22908268

[11] Su XY Zou X Chen QZ Zeng YH Shao Y He BC. Follicle-stimulating hormone β-subunit potentiates bone morphogenetic protein 9-induced osteogenic differentiation in mouse embryonic fibroblasts. J Cell Biochem (2017) 118:1792–802. doi: 10.1002/jcb.25849 27996168

[12] Gallagher CM Moonga BS Kovach JS. Cadmium, follicle-stimulating hormone, and effects on bone in women age 42-60 years, NHANES III. Environ Res (2010) 110:105–11. doi: 10.1016/j.envres.2009.09.012 19875111

[13] Sowers MR Greendale GA Bondarenko I Finkelstein JS Cauley JA Neer RM. Endogenous hormones and bone turnover markers in pre- and perimenopausal women: SWAN. Osteoporos Int (2003) 14:191–7. doi: 10.1007/s00198-002-1329-4 12730778

[14] Sowers MFR Jannausch M McConnell D Little R Greendale GA Finkelstein JS. Hormone predictors of bone mineral density changes during the menopausal transition. J Clin Endocrinol Metab (2006) 91:1261–7. doi: 10.1210/jc.2005-1836 16403818

[15] Rendina D Gianfrancesco F De Filippo G Merlotti D Esposito T Mingione A. FSHR gene polymorphisms influence bone mineral density and bone turnover in postmenopausal women. Eur J Endocrinol (2010) 163:165–72. doi: 10.1530/EJE-10-0043 20335500

[16] Devleta B Adem B Senada S. Hypergonadotropic amenorrhea and bone density: New approach to an old problem. J Bone Miner Metab (2004) 22:360–4. doi: 10.1007/s00774-004-0495-1 15221495

[17] Kawai H Furuhashi M Suganuma N. Serum follicle-stimulating hormone level is a predictor of bone mineral density in patients with hormone replacement therapy. Arch Gynecol Obstet (2004) 269:192–5. doi: 10.1007/s00404-003-0532-7 13680264

[18] Drake MT McCready LK Hoey KA Atkinson EJ Khosla S. Effects of suppression of follicle-stimulating hormone secretion on bone resorption markers in postmenopausal women. J Clin Endocrinol Metab (2010) 95:5063–8. doi: 10.1210/jc.2010-1103 PMC2968737 20610587

[19] Omodei U Mazziotti G Donarini G Gola M Guella V Pagani F. Effects of recombinant follicle-stimulating hormone on bone turnover markers in infertile women undergoing *in vitro* fertilization procedure. J Clin Endocrinol Metab (2013) 98:330–6. doi: 10.1210/jc.2012-2778 23093489

[20] Karim N MacDonald D Dolan AL Fogelman I Wierzbicki AS Hampson G. The relationship between gonadotrophins, gonadal hormones and bone mass in men. Clin Endocrinol (Oxf) (2008) 68:94–101. doi: 10.1111/j.1365-2265.2007.03005.x 17760881

[21] Ferlin A Schipilliti M Vinanzi C Garolla A Di Mambro A Selice R. Bone mass in subjects with klinefelter syndrome: Role of testosterone levels and androgen receptor gene cag polymorphism. J Clin Endocrinol Metab (2011) 96(4):E739–45. doi: 10.1210/jc.2010-1878 21270324

[22] Clarke NW McCLURE J George NJR. Morphometric evidence for bone resorption and replacement in prostate cancer. Br J Urol (1991) 68:74–80. doi: 10.1111/j.1464-410X.1991.tb15260.x 1873694

[23] Wu KC Ewing SK Li X Sigurðsson S Guðnason V Kado DM. FSH level and changes in bone mass and body composition in older women and men. J Clin Endocrinol Metab (2021) 106:2876–89. doi: 10.1210/clinem/dgab481 PMC8475206 34212197

[24] Antonio L Priskorn L Olesen IA Petersen JH Vanderschueren D Jørgensen N. High serum FSH is not a risk factor for low bone mineral density in infertile men. Bone (2020) 136:115366. doi: 10.1016/j.bone.2020.115366 32304878

[25] Juel Mortensen L Lorenzen M Jørgensen N Andersson AM Nielsen JE Petersen LI. Possible link between FSH and RANKL release from adipocytes in men with impaired gonadal function including klinefelter syndrome. Bone (2019) 123:103–14. doi: 10.1016/j.bone.2019.03.022 30914274

[26] Uihlein AV Finkelstein JS Lee H Leder BZ. FSH suppression does not affect bone turnover in eugonadal men. J Clin Endocrinol Metab (2014) 99:2510–5. doi: 10.1210/jc.2013-3246 PMC4079307 24646101

[27] Ng Tang Fui M Hoermann R Bracken K Handelsman DJ Inder WJ Stuckey BGA. Effect of testosterone treatment on bone microarchitecture and bone mineral density in men: a two-year RCT. J Clin Endocrinol Metab (2021) 106(8):e3143–58. doi: 10.1210/clinem/dgab149 33693907

[28] Jayasena C Anderson RA Llahana S Barth JH MacKenzie F Wilkes S. Society for endocrinology guidelines for testosterone replacement therapy in male hypogonadism. Clin Endocrinol (Oxf) (2021) 96(2):200–19. doi: 10.1111/cen.14633 34811785

[29] Federici S Goggi G Quinton R Giovanelli L Persani L Cangiano B. New and consolidated therapeutic options for pubertal induction in hypogonadism: in-depth review of the literature. Endocr Rev (2021) 1:bnab043. doi: 10.1210/endrev/bnab043 34864951

[30] Husten CG. How should we define light or intermittent smoking? does it matter? Nicotine Tob Res (2009) 11:111–21. doi: 10.1093/ntr/ntp010 PMC2658911 19246425

[31] Genant HK Wu CY van Kuijk C Nevitt MC. Vertebral fracture assessment using a semiquantitative technique. J Bone Miner Res (1993) 8:1137–48. doi: 10.1002/jbmr.5650080915 8237484

[32] Vermeulen A Verdonck L Kaufman JM. A critical evaluation of simple methods for the estimation of free testosterone in serum. J Clin Endocrinol Metab (1999) 84:3666–72. doi: 10.1210/jcem.84.10.6079 10523012

[33] Tirabassi G Delli Muti N Gioia A Biagioli A Lenzi A Balercia G. Effects of testosterone replacement therapy on bone metabolism in male post-surgical hypogonadotropic hypogonadism: Focus on the role of androgen receptor CAG polymorphism. J Endocrinol Invest (2014) 37:393–400. doi: 10.1007/s40618-014-0052-2 24458833

[34] Zaidi M Turner CH Canalis E Pacifici R Sun L Iqbal J. Bone loss or lost bone: Rationale and recommendations for the diagnosis and treatment of early postmenopausal bone loss. Curr Osteoporosis Rep (2009) 7:118–26. doi: 10.1007/s11914-009-0021-4 19968915

[35] Randolph JF Sowers M Gold EB Mohr BA Luborsky J Santoro N. Reproductive hormones in the early menopausal transition: Relationship to ethnicity, body size, and menopausal status. J Clin Endocrinol Metab (2003) 88:1516–22. doi: 10.1210/jc.2002-020777 12679432

[36] Bojesen A Birkebæk N Kristensen K Heickendorff L Mosekilde L Christiansen JS. Bone mineral density in klinefelter syndrome is reduced and primarily determined by muscle strength and resorptive markers, but not directly by testosterone. Osteoporos Int (2011) 22:1441–50. doi: 10.1007/s00198-010-1354-7 20658127

[37] Ferlin A Selice R Di Mambro A Ghezzi M Di Nisio A Caretta N. Role of vitamin d levels and vitamin d supplementation on bone mineral density in klinefelter syndrome. Osteoporos Int (2015) 26:2193–202. doi: 10.1007/s00198-015-3136-8 25963234

[38] Di Nisio A De Toni L Rocca MS Ghezzi M Selice R Taglialavoro G. Negative association between sclerostin and INSL3 in isolated human osteocytes and in klinefelter syndrome: New hints for testis-bone crosstalk. J Clin Endocrinol Metab (2018) 103:2033–41. doi: 10.1210/jc.2017-02762 29452406

[39] Bojesen A Hertz JM Gravholt CH. Genotype and phenotype in klinefelter syndrome - impact of androgen receptor polymorphism and skewed X inactivation. Int J Androl (2011) 34(6 Pt 2):e642–8. doi: 10.1111/j.1365-2605.2011.01223.x 21977989

[40] Casimiro I Sam S Brady MJ. Endocrine implications of bariatric surgery: a review on the intersection between incretins, bone, and sex hormones. Physiol Rep (2019) 7(10):e14111. doi: 10.14814/phy2.14111 31134746 PMC6536581

[41] Eller-Vainicher C Falchetti A Gennari L Cairoli E Bertoldo F Vescini F. Diagnosis of endocrine disease: Evaluation of bone fragility in endocrine disorders. Eur J Endocrinol (2019) 180:R213–32. doi: 10.1530/EJE-18-0991 31042675

[42] Chiodini I Catalano A Gennari L Gaudio A. Osteoporosis and fragility fractures in type 2 diabetes. J Diabetes Res (2020) 2020:9342696. doi: 10.1155/2020/9342696 PMC7378603 32733970

[43] Bonomi M Vezzoli V Krausz C Guizzardi F Vezzani S Simoni M. Characteristics of a nationwide cohort of patients presenting with isolated hypogonadotropic hypogonadism (IHH). Eur J Endocrinol (2018) 178:23–32. doi: 10.1530/EJE-17-0065 28882981

[44] Cangiano B Duminuco P Vezzoli V Guizzardi F Chiodini I Corona G. Evidence for a common genetic origin of classic and milder adult-onset forms of isolated hypogonadotropic hypogonadism. J Clin Med (2019) 8:126. doi: 10.3390/jcm8010126 PMC6352096 30669598

[45] Johnell O Kanis JA. An estimate of the worldwide prevalence and disability associated with osteoporotic fractures. Osteoporos Int (2006) 17:1726–33. doi: 10.1007/s00198-006-0172-4 16983459

[46] Gera S Sant D Haider S Korkmaz F Kuo TC Mathew M. First-in-class humanized FSH blocking antibody targets bone and fat. Proc Natl Acad Sci USA (2020) 117:28971–9. doi: 10.1073/pnas.2014588117 PMC7682550 33127753

[47] Xiong J Kang SS Wang Z Liu X Kuo TC Korkmaz F. FSH blockade improves cognition in mice with alzheimer’s disease. Nature (2022) 603(7901):470–6. doi: 10.1038/s41586-022-04463-0 PMC9940301 35236988

[48] Geng W Yan X Du H Cui J Li L Chen F. Immunization with FSHβ fusion protein antigen prevents bone loss in a rat ovariectomy-induced osteoporosis model. Biochem Biophys Res Commun (2013) 434:280–6. doi: 10.1016/j.bbrc.2013.02.116 23537645

